# A phenomenological study of business graduates’ employment experiences in the changing economy

**DOI:** 10.1186/s12651-018-0238-8

**Published:** 2018-03-19

**Authors:** Throy Alexander Campbell

**Affiliations:** 1 Business & Information Systems, McKee B & T Building, 7000 Adventist Blvd NW, Huntsville, AL 35896 USA; 2 Learning Innovation and Networked Knowledge Research Lab, The University of Texas at Arlington, Arlington, TX USA

**Keywords:** Labor market, Career paths, Automation, Higher education, J21, J23, J40, J60

## Abstract

This study explores the perspectives of business college graduates, how technology has shaped the structures of their jobs, and the role of non-technical skills as they navigate the changing career path. Three overlapping themes emerged from the data analysis: (1) influence of increased technology capabilities on job structures and careers; (2) participation in job-related training and formal education as means of adapting to the new work environment; and (3) the role of non-technical skills in the workplace amidst the intensification of technology change. This research provides higher education practitioners and labor market researchers qualitative perspectives on work structure changes.

## Introduction

Business schools are tasked with the responsibility to train the next generation of managers and leaders, equipped with the skill sets to continuously improve business operations (Glen et al. 2014; Gordon and Howell 1959; Simon 1967). However, there is a widening gap between graduates’ skills and employer expectations (Bailey and Mitchell 2006; McGuinness and Sloane 2011). This disconnect calls for business schools to hire instructors with business world experience (Harrison et al. 2007; Simon 1967; Van de Ven and Johnson 2006) and to adopt curricula that provide learning in non-technical skills (soft skills) as a complement to current functional areas, such as accounting, finance, marketing, and information systems (Glen et al. 2014; Desai et al. 2016). The changing business environment in relation to the business school curricula was assessed by Simon (1967) who put forward that business schools should develop pedagogies concerned with the “what ought to be” instead of “the what is.” Over the last decades, job automation due to advancement in machine learning and artificial intelligence has intensified (Autor et al. 2003; Frey and Osborne 2013). The unknown is how has technology influence business professions, which traditionally are not overly influenced by technology change.

The changing of job structures due to the advancement of technology was researched and explained by Autor et al. in their novel task model (2003). The researchers utilized occupational and industry information from the *Dictionary of Occupational Titles* (DOT) and the *Current Population Survey* (CPS), which distinguish between the task input per job (units of work) and the type of skills (worker capability) required to operationalize the tasks. They identified changes over time in the demand for non-routine versus routine tasks. Frey and Osborne (2013) later developed a predictive model that considered the latest development in big-data algorithms, machine intelligence, and robotics. The new method helps predict the probability of job computerization per industry, city, and country. A common finding of both bodies of research was that highly-skilled workers in technical areas (hard skills) are better positioned if they possess unique non-technical skills such as problem-solving, emotional regulation, politicking, moral sensibilities, and communication (Neubert et al. 2015).

The task model and job computerization method have provided details on the future prospect of jobs per task composition, but there were certain limitations. Autor et al. noted that the *Dictionary of Occupational Titles* “includes a limited sampling of occupations (partly in the service sector), imprecise definitions of measured constructs, and omission of important skills” (2003, p. 1293). Frey and Osborne (2013) also admitted that their model on the probability of occupation automation estimates does not account for variations within occupations.

The existing research, mainly quantitative, have not focused specifically on business professions, hence, the need to explore these areas. This study uses a qualitative approach to source the real-life perspectives of business workers. The overarching goal is to present business graduates’ descriptions of their careers and the skill adjustment they have made due to the influence of technology. This research serves to inform higher education practitioners and labor economics researchers.

The next sections of this paper provide a more indepth background of the influence of technology on job structures, the role of non-technical skills amidst technological advancements, and business school response to technological changes in the workplace.

## Background

### Job structure and technological change

To what extent human position in the labor market is threatened by the advancement of workplace technology remains an area of inquiry for labor economists. The novel task model put forth by Autor (2015), Autor and Price (2003) and Autor et al. (2003) which later influenced (Frey and Osborne 2013) job computerization predictive methodology, help to measure and estimate future skill demands. In their task model, Autor et al. matched data on task requirements and skill requirements by using data from the *Dictionary of Occupational Titles* (DOT) and the *Current Population Survey* (CPS). They identified consistent task inputs per occupation and industry between 1960 and 1998. The study was later extended to include data through 2010 (Autor and Price 2003). The authors posited that their new methodology differs from previous approaches, as it enables nuanced analysis per the type of task performed in specific jobs rather than the average educational credentials of workers within a particular occupation or industry (Autor et al. 1998; Berman et al. 1998; Bresnahan et al. 2002).

The model defines the different tasks performed by computers and humans. Eventually, computers were able to execute clear, repetitive, routine instructions, but over time, evolved to manage greater volumes of data at high speed. This resulted in the automation of repetitive manual labor across manufacturing and service industries (i.e., production lines and bank teller services). On the other hand, tasks specific to humans (at least in the foreseeable future—discussed below as questioned by the novel automation literature) are both manual and cognitive; not readily defined and non-routine; and requires social intelligence and adaptability (Pinker and Bradshaw 1997; Polanyi 1966). Therefore, computer technology is more able to substitute for low-skill level labor in carrying out manual routine task, as more of a complement to high-skilled, non-routine complex/cognitive labor. These advancements in workplace technology, coupled with a drastic reduction in computer prices have caused wholesale adoptions by employers to replace more expensive labor. This increased labor input and enabled highly-skilled/educated workers to focus on workplace efficiency and innovation. Also, humans are now required to be adaptable, communicate effectively, and to collaborate in problem-solving teams.

In both the original and follow-up analyses (see Autor and Price 2003; Autor et al. 2003) details are provided on the different task categories and skill requirements. At one end of the skill spectrum is the ‘abstract tasks’ which require more human interaction such as creativity, collaboration, intuition, and persuasion. These tasks are found in professional, leadership, technical and analytic fields. Individuals in these positions hold college degrees, are more likely to adjust to advancement in computing, and are at the top of the earnings scale. At the other end of the spectrum are the ‘manual tasks’ which require “situational adaptability, visual and language recognition, and in-person interaction” (Autor and Price 2003, pp. 2–3). These tasks are found in jobs such as truck driving, housekeeping, and meal preparation. These jobs require low levels of training and are at the lowest end of the salary scale. The researchers, therefore, posit that computerization contributes to the polarization of jobs by pushing to the lower end of the job scale task intensive, low-paid jobs, and at the top of the scale are the high-skilled, high-paid occupations. Hence, individuals with mid-level skills are being forced either to work in jobs at the lower end of the scale or undergo further training/schooling to position for jobs at the higher end of the scale.

While the task model outlines how job structures have changed, Frey and Osborne (2013) model predicts changes for the future. They incorporated developments in digital connectivity, big data algorithms, machine learning, and artificial intelligence, which enabled further computerization of non-routine tasks previously categorized for humans. Previously complex tasks can now be decoded into simple routinized instructions. The researchers utilized the updated online version of the DOT: Occupational Information Network (O*NET) database that contains standardized, measurable variables on U.S. occupations. These variables reflect the changing nature of jobs and capture a diverse range of skill sets such as “originality, service orientation, manual dexterity, and gross body coordination.” The variables were then rated by co-researchers and experts in technical fields, based on whether they were automatable or not. The fundamental question was: “Can the tasks of this job be sufficiently specified, conditional on the availability of big data, to be performed by state of the art computer-controlled equipment?” A probabilistic classification algorithm was then used to learn the relationships between the labels of automatability and the O⋆NET variables. The main findings affirmed the results of the task model, but also suggested a wider range of routine and non-routine tasks are likely to be computerized in the future.

### Human skills and technological change

The task model promoted by Autor et al. highlighted the role of humans’ managerial and interpersonal skills required under the abstract task category, and the “situational adaptability, visual and language recognition, and in-person interaction” (Autor and Price 2003, p. 2) skills required under the manual task category. Frey and Osborne (2013) also showed that human skills such as negotiation, persuasion, and social intelligence remain as bottlenecks to be overcome for full automation of certain jobs. Hence, human roles remain viable in the foreseeable labor market. Geoffrey Colvin (2015), author of *Humans Are Underrated*, argues the role of humans in the workplace: “Some tasks require a high level of perception and manipulation, where people can see and respond to circumstances in ways that robots and computers cannot. [Also], jobs requiring creativity and social skills are not susceptible to automation.” The results from a recent National Association of Colleges and Employers (NACE) Job Outlook 2016 survey of employers found that 80% of employers still desire leadership abilities of its employees. Other on-demand skill sets include the ability to work in a team structure, communication, problem-solving, and strong work ethics.

These studies on the type of skills graduates possess and the match between these skills and employer requirements allude to the traditional debate on the value of college liberal arts degree programs versus the applied sciences (Giles and Drewes 2002). Recent social science research affirms the above mentioned labor economics research that those who work in technical areas are now being required to have complex and collaborative problem-solving skills including emotional regulation, politicking, moral sensibilities, and communication (soft skills) (Giles and Drewes 2002; Neubert et al. 2015). Ironically, the growth in demand for technical expertise (hard skills) within tech-intensive industries seems to have increased the demands for complementary liberal arts proficiency (Pichler and Beenen 2014).

### Business school response

Do business graduates experience the shift in workplace skill demands as those in high technological professions? The research noted thus far points to the significance of non-technical skills, as also affirmed by recent surveys of business leaders. For example, the *National Surveys of Business and Nonprofit Leaders and Current College Students*, conducted by the Association of American Colleges and Universities found that more than 75% of employers emphasized the need for critical thinking, complex problem solving, written and oral communication, and applied knowledge in real world settings (Hart Research Associate 2015). These skills or attributes were also highly ranked by employers when looking at candidates’ resumes, as reported by the National Association of Colleges and Employers’ (NACE) Job Outlook 2016 survey.

An appropriate question, then, is how higher education institutions, particularly business schools, are responding to this demand by the future employers of their students. Empirical research has examined the perspectives of business schools’ faculty on how non-technical skills are being incorporated into the curriculum. While the Association to Advance Collegiate Schools of Business (AACSB) has called for increased focus on non-technical skills, particularly critical thinking (CT), it was found that business school faculty members differ on their perception of what CT and other non-technical skills measure, causing ambiguity across the various curricula (Desai et al. 2016). Emerging research suggests the redesigning of the curriculum requires a move away from the debate on the balance of functional areas such as accounting, finance, management, marketing, and information systems (Desai et al. 2016; Dyer et al. 2011; Glen et al. 2014; Waddock and Lozano 2013) to means of developing non-technical and other transferable skills for increasing technological and boundary-less organizations (Autor et al. 2003; Frey and Osborne 2013; Neubert et al. 2015).

This study is unique as little is known about job structural changes from a real-life-perspective. Also, the focus is on business school graduates on their work experiences in recent years. Through open-ended questions, this study seeks to understand the “lived experiences” of college graduates in technology-intensive business environments, as to how technology has shaped the nature of work and the role of non-technological skills for a successful career path.

The following research questions will be addressed:

1. How do business graduates perceive their career experiences as influenced by the advancement of information communication technologies in the workplace? How do their education backgrounds such as field of study and degree level prepare them for changes?
2. To what extent do business graduates perceive computerization as a threat or not to their careers? What career and educational adjustment are they making to ensure their employability in the future?
3. What is the role of non-technical (soft skills) in today’s workplace? Do non-technical skills benefit business graduates at different job levels?

## Methods

This study adopted a phenomenological research methodology and employed an interviewing method of data collection (Smith et al. 2009). This methodology has evolved as a means for exploring participants’ perspectives of their interactions with other people and social situations (Crotty 1998). The main goal is to provide a first-person experience. Phenomenology requires the researcher to place his interpretations aside and present the raw descriptions provided by the subjects. This, admittedly, is limited as it seeks to isolate the researcher’s cultural impositions and at the same time not be suspicious of the participants’ interpretation of their experiences (Crotty 1998). Nevertheless, as the researcher is responsible for reporting selected themes of the participants’ descriptions, it is noticeable that phenomenology has a degree of objectivity—the selection of objects of the subjects’ experiences (Creswell 2013; Maxwell 2012). The method employed, as described below, was geared towards collecting and analyzing data and in so doing minimized the imposition of the researcher’s presumptions.

### Data collection

Approval was obtained from the Institutional Research Board at the authors’ university to obtain graduates’ email addresses from the College of Business’ alumni department. An invitation to the study was emailed to graduates with their first degree in business fields: accounting, finance, marketing, and information systems (these business areas of work are identified to be directly influenced by technology). Only ten participants were interviewed (three of which subsequently completed their master’s degree also in business fields) as ongoing analysis revealed similar themes with no variedly new information (Creswell 2013; Maxwell 2012). This homogeneity in the participants’ responses, which were outside of the researcher’s control, provided an in-depth understanding of the group’s experiences. After the graduates had provided written informed consent and completed a demographic survey, a semi-structured interview was conducted via Zoom, a web and video conference service. Each interview lasted for approximately 45 min. The interviews were semi-structured with references to the following three questions: (1) Describe your career path over the last 5 years. What are the trends in relation to how jobs are structured and are changing? (2) How have you adjusted to technological change? (3) How do you perceive the role of non-technical skills in a technologically intensive work environment? The overarching goal was to learn about graduates’ experiences in relation to technological changes in the workplace. The interviews were audio recorded via the Zoom system and later downloaded and stored securely under pseudonyms. The recordings were later transcribed verbatim by professional transcriptionists. The interviewers (authors) wrote a brief reflective memo after each interview as a first impression. Table 1 provides a listing of the demographic characteristics and basic work information of the participants.

**Table 1:** Participants’ demographic and basic work characteristics

Pseudonym	First degree	Graduate degree	Industry	Year of degree	Gender	Age
Ben	Accounting	None	Accounting	1995	Male	42
Bill	Info. Systems	None	Info. Systems	2003	Male	38
Gina	Accounting	None	Healthcare	1997	Female	52
Mark	Finance	MBA	Finance	1999	Male	48
Mathew	Accounting	None	Accounting	2004	Male	50
Mou	Administration	Info. Systems	Healthcare	1997	Male	54
Roger	Accounting	None	Accounting	2000	Male	48
Sean	Info. Systems	None	Agriculture	2002	Male	51
Tresha	Accounting	Accounting	Finance	2008	Female	28
Victor	Info. Systems	None	Info. Systems	2010	Male	32

### Data analysis

Interpretative phenomenological analysis (IPA) is a cyclical process which includes several iterative stages. An emergent analysis strategy was used to abstract explicit and implicit themes from the interview transcripts. Throughout the analysis, care was taken to use only narratives that maintain the meaning of the original text.

A computer-assisted qualitative data analysis (CAQDAS) program, MAXQDA 12, was employed. However, it is important to emphasize that the software does not assist with code selection and decisions on findings; it is merely an organization tool. The stages of analysis included repeated reading of the text to identify the pure perspectives of the participants (coding process); making reflective comments and memos on a line by line basis if necessary to identify essential components of the text; mapping interrelationships and patterns between data segments; and tabulating key themes.

MAXQDA 12 enables hierarchical organization of identified codes and the automatic retrieval of all data associated with the codes. The researchers created and linked reflective memos to selected segments of the data. From the list of codes, the researchers were able to search, highlight, group, and modify codes across transcripts. The researchers were able to click on a particular code or group of codes and simultaneously identify memos and original segments of the data. Furthermore, the software enabled the researchers to attach participants’ demographic, work, and education characteristics for cross-tabulations, and also quantify codes and key data segments. The analysis was conducted separately by the authors who, then, discussed and chose common themes that emerged.

### Validity/trustworthiness

The IPA, like other qualitative methods of analysis, undergoes constructive criticisms on the validity of the processes. The main concern is the ability of the researchers to not impose their biases and values during the analysis stage of the study. Several measures were taken to preserve the pureness of the participants’ perspectives (Creswell 2013; Maxwell 2012): (1) participants were able to freely express perspectives of their experiences. Furthermore, the participants’ perspectives were directly quoted, and when observations were made by the author, the referred comments were also directly quoted. (2) The author kept a rigorous audit trail of reflective memos throughout the data analysis, to isolate/bracket his perspectives from those of the participants (Creswell 2013). (3) Member checking was conducted by sharing analytical categories, findings, and interpretations of the findings with participants via email, who were able to suggest changes and clarifications. (4) An experienced qualitative researcher was asked to review the interview transcripts, analytical memos and notes, and the findings of the study as a means of confirming the researchers’ analyses.

## Results and discussion

Previous research highlights an intensifying interplay between human capabilities and the capacity of workplace computing. Analysis of the college graduates’ perspectives of their work experiences provides unique insights into the changing phenomenon of work. Three overlapping themes emerged from the data analysis: (1) influence of increase technology capabilities on job structures and careers; (2) participation in job-related training and the role of formal education as means of adapting to the new work environment; and (3) the role of humans in the workplace amidst the intensification of technology change.

### Theme 1: Influence of increase technology capabilities on job structures and careers

The participants were employed in various professions, including information technology, financial consulting, agriculture marketing, and healthcare management. As put forward in previous studies, the college graduates have all experienced structural changes in their jobs. In other words, technology has impacted the type of task they execute and the type of skills required. While new technical skills are required of the college graduates, the human element remains essential. The participants also mentioned that with new technology comes new regulations regarding information privacy. In general, the participants showed great adaptability, but there has been some resistance, particularly from older workers.

First, all the graduates expressed that “growth in virtual networking,” “digital connectivity,” and “data availability” have ramifications for their careers. Victor, an IT network manager who works for different clients virtually from his home-office, noted, “I can do my job and collaborate with peers and clients from anywhere, but I often travel to clients’ location when necessary.” He suggested that visualization technology has not only impacted where he works but also how he works.

The way that virtualization technology has exploded since it was introduced has changed things a lot, for instance, at my company we recently moved from a 10,000 square foot datacenter into 1000 square foot kolo with about 20 cages. Things are changing pretty quickly!

Victor further expressed that the technical skills required in his area of work continue to change; however, he is very optimistic about the future of his career as he can adapt and learn new skills:

There’s a lot of new technology which I am able to learn and come into [the labor market] with skills that others don’t have. For instance, with load balancers, there are different firewall companies have come out with layer seven firewalls and things of that nature. So, it helped me a lot.

Mark, a manager within a financial firm, expressed somewhat similar perspectives as Victor. He described how over time the technology he uses has changed, but with positive implications for work and his career:

When I first started out my career a lot of data was tracked in either an Access database or an Excel spreadsheet, which was very flawed and limited in how you can share data… The technology that I’ve used over time has gone literally from spreadsheets to more comprehensive databases and business intelligence tools. I’ve obtained training in Cognos, Oracle, Business-Logics, and … PPW warehouse which is a SAP business intelligence software. This enables one to determine trends, opportunities, and potential risks to your business.

These perspectives characterized by Victor and Mark, as IT and financial professionals, respectively, are indicative of Autor et al. (2013) research on the change in job content over time, influenced by technology, and the complementarity of changing job tasks with highly-skilled individuals. These graduates shared how they were able to undergo training and adapt to new technology (will be further discussed in the subsection below), but it is evident that as technology advanced the task content and skill requirements changed; college-educated workers seem to possess the flexibility and creativity to navigate. Virtualization also requires skills by which workers can collaborate and impact their businesses across both physical and virtual environments.

Second, while the new technology is presenting businesses and individuals with new tools, a critical concern expressed by eight of the ten graduates, interviewed, more so by those in healthcare and financing, is the issue of information privacy. According to Mou, a healthcare IT professional, “This impacts the means of data collection, storage, and usage. Considering that big-data is being incorporated into decision making, the increase connectivity and software usage have come with an increased security risk.” Regarding the influence of technology on the structure of work, in his medical practice, there have been mixed results: “Technology continues to transform how work is conducted. There has been better metrics on medicine and digitization of medical records, which has improved workplace efficiency, but the focus has shifted to system maintenance.” He is capable of managing the new IT changes; however, Mou noted disruption over time among healthcare professionals: “Some of the doctors refused to go to the digitization; the younger doctors were receptive, but some of the older ones just retired.” It can be inferred from Mou’s observation that older workers who may have completed their college education years ago, may have found it difficult to make the necessary technical adjustments.

The other participants further emphasized that security issues are shaping the way they do work. Businesses are investing significantly in cyber security as it can have financial consequences. Ben noted, “it cannot be overstated enough regarding the security of clients’ files and their digital property.” Ben further advised: “college graduates entering the workforce need to understand that this is a very real thing. A security breach can have serious financial ramifications. It can also become a legal issue.” Tresha, a financial auditor, gave a different perspective, she noted that the advancement of technology had created the need for IT auditor to work along with general business auditors:

For each audit, we have an IT auditor because we’re not just looking at paper-based documents anymore. But now that there are all these systems in place, I have been forced to understand how to process private materials. It is important to know who has access, and what type of access they have. Is it possible to override or bypass certain protocols and rules in the system? IT audit includes data integrity, especially in the financial services industry.

Tresha’s perspective on how the increased usage of network technology has created new jobs and career opportunities affirms (Frey and Osborne 2013) predictions on the growth in a new type of jobs (i.e., security analysts, cloud architects, and data scientists). This was elaborated in a recent Australian study on *Tomorrow’s Digitally Enabled Workforce*: “The cybersecurity professional is likely to be accompanied by professionals, with expertise in the management of online identities and social media, who protect people’s interests.” (Hajkowicz et al. 2016, p. 82).

Finally, Sean, an information systems manager, suggested that with technological advancement, his expertise has become of higher importance for his firm: “The emergence of big-data capabilities shapes the competitiveness of firms as a whole, but requires pro-activeness.” He further noted:

Every piece of information that I want to know has contained dataset somewhere. Today, those datasets are walled off, and they’re difficult to integrate. But slowly but surely, my company and other companies are merging those datasets to give bigger pictures of the market”. Data is never going to be perfect, and one’s ability to manipulate the data determines “whether or not you’re successful in your project.

The graduates acknowledged changes to job structures and alluded to the type of skills that will be required. In the next subsection, information on the type of training the graduates have undergone to enhance their employability in the changing landscape of work is presented.

### Theme 2: Participation in job-related training and the role of formal education

Research has utilized longitudinal data sets to track job content change and to predict the probability of future computerization (Autor and Price 2003; Autor et al. 2003; Frey and Osborne 2013), but uncertainty lingers. The graduates who participated in this study have witnessed changing technological trends in recent times and provide real life experiences. The brief survey which was administered in conjunction with the open-ended interviews enables the researcher to somewhat quantify reasons for the participants’ involvement in job-related training. At least seven of the graduates indicated that they were involved to gain further education before beginning a career; to increase opportunities for promotion, advancement or higher salary; and as required or expected by employers. Noticeably, none of the graduates indicated that their intent was to change their academic or occupational field or to prepare for graduate school or further education. Two of the graduates also participated for licensure or certification. While these numbers cannot be generalized to the wider population, they are indicative of the graduates’ need to acquire new skill sets in addition to the ones they initially obtained through a college education. While the adequacy of education attainment is argued, it is reasonable to suggest that further education is necessary to stay proficient in the changing workplace. A question of continued debate is whether current higher education programs are adequately preparing graduates for the changes in the labor market (Giles and Drewes 2002).

The graduates also provided details on how their formal education have initially prepared them for their jobs and the importance of additional training. Roger, like others, mentioned that his college degree “opened the door of employment.” Victor posited that his degree in information systems taught him how to think critically:

As far as the networking profession goes, you always have to pay attention to details. And it’s always something very minute that you don’t think about. So, from that standpoint [of college education] I had to understand what I was doing the math, programming…, and static classes. It made me a better person.

Ben mentioned that “outside of the technical aspect, there is a maturing that takes place in one’s academics and personality when going through higher education. This is key to being more marketable in the workplace.” Bill noted that his degree in Information Systems prepared him holistically:

Not all my classes were computer [sciences], some were in marketing and management. Being able to see the two sides of the coin. I mean, the whole person of an information technologist, is to be a liaison between the machine and upper management. And so being able to understand the business side of things [helps] to accomplish the business goals.

Bill explained further: “I still have to maintain my certifications. Future graduates will be challenged to get a certification such as Cisco or Network Plus or A Plus. These programs have skyrocketed [my] career.” Gina further emphasized the importance of certifications as an Accountant: “Well, I think, if I went back and got my MBA, it would help in the workplace. But I think that my CPA license is more valuable… My director doesn’t have an MBA, but he’s the controller of this huge corporation.” Mou also noted: I continually take Coursera and Udacity classes. Victor went a step further by setting-up “Cisco lab at home; I have volunteers from various vendors, F5, CITRIX, and Riverbed. I have two different vendors, firewalls that I’m running as my gateway to the internet. So I get … familiar with it.”

The graduates spoke less about returning to higher education institutions to pursue advanced degree programs. This is suggestive of a shift to non-traditional means of education delivery facilitated by technology. Online certification programs have gained prominence. The relationship between education and technology is also changing. Workers are demanding more flexible and specific kinds of education and training; educators are tailoring their curriculum and means of delivery to meet the needs of non-traditional students and employers are investing in work training programs in collaboration with higher education institutions to fill the changing occupational needs.

Noticeably, the graduates also highlighted the importance of non-technical training. While most graduates in this study participated in workplace-related training in their occupational field, they were involved in management or supervisory and general professional training (e.g., public speaking, business writing). Furthermore, most of the graduates highlighted the importance of proper communication skills in the changing workplace. Bill’s perspective is representative of the others:

Non-technical skills such as the ability to communicate effectively is an example. As an IT worker, if you overload a coworker with a bunch of jargons, you’re not effectively communicating, which can lead to conflict in the workplace. Also, when you can take technology and translate into dollars and cents …it helps with decision making. So it’s critical.

The next subsection highlights the role of humans amidst the technological changes in the workplace. Non-technical skills, such as the ability to communicate effectively the technical aspects of one’s job to decision makers, is further discussed below.

### Theme 3: The role of humans amidst the intensification of technological

The expansion of digitization and usage of algorithms on large data sets have advanced the functionality of workplace technology. In recent times the scientific community has been optimistic about the capability of computers to perform more rational (cognitive) tasks (Brynjolfsson and McAfee 2011; Veres et al. 2011). However, research still suggests that highly-skilled individuals, for example those employed in leadership roles, who possess critical thinking, problem-solving, collaborating skills, are likely to be in great demand in the foreseeable future (Frey and Osborne 2013). The perspectives provided by the college graduates considered in this study contribute to the scholarly debate.

The graduates unanimously highlight the importance of strong communication skills. According to Bill:

Technology has drastically changed skills. Virtualization as an example, a little over 10 years ago would never have been considered in an enterprise environment running critical systems. Now it’s the norm. However, the ability to communicate is critical … what is unique about me is that I can communicate technology issues in ways that most people cannot. Even other IT Professionals, who may have more certifications than I do, may not be able to have the type of rapport that I can usually build almost instantly with our customers.

Victor also emphasized that as a human worker, he can interact and communicate with clients in ways that a robot cannot:

As the IT expert, I have to be able to understand what clients need for their business to function efficiently. For instance, a client may ask for a more balanced server, ‘I needed a server that redirects to the servers on the backend.’ There is no way a robot or machine can interpret that and explain back to an individual the best options based on their business operations.

The graduates provided additional meaningful perspectives on the role of humans versus technology in the workplace. Tresha argued that being analytical is a key skill in the financial industry: “The ability to analyze data quickly and provide clients with real-time options is critical.” According to Roger,” technology can assist you in making a decision, but at the end of the day, in my particular line of work, it has to be a person making that decision based on the data obtained.” Mou added, “I don’t know where that next part of artificial intelligence is going, but ultimately the onus of utilizing the technology falls on people like you and the individual to utilize it best and to be able to make the best decisions…the technology is just a tool. Teresa further noted: “As the systems get more sophisticated. I think we have to do more training to take advantage of employment opportunities. Today, a first-year associate is now doing what a senior associate with more experience has done in the past. Each level has risen one level higher.”

The findings of this study affirm previous research on the likely employment outcomes of the highly-skilled/educated workers, whereas, the chance of human obliteration from the workforce is not foreseeable (Autor 2015; Autor and Price 2003; Autor et al. 2003; Neubert et al. 2015). The human ability to perceive, manipulate, recreate, collaborate, and communicate are a few of the skills that remain as core competencies of work. However, the responsibility is on humans to position themselves through further work-related training and higher education to navigate the digitized and changing landscape of work.

## Conclusion

Computer technology will continue to be pervasive in the workplace with increasing capacity to substitute and/or complement the role of human workers. Yet, this paper highlights the experiences of business graduates who, in general, are positioned to take advantage of the new employment opportunities that emerge in the changing work environment. A key takeaway from this study is that while the graduates acknowledge that their formal education opened doors for their initial employment, they emphasized the importance of continued education or job training to learn new skills as required by the changing job structures. As in previous research, the graduates posited that well defined rudimentary tasks have been mostly automated. This has enabled highly skilled workers to focus on ill-defined tasks which require human abilities to negotiate and make decisions to serve client demands. The graduates not only expressed the need to learn advanced technical skills, but emphasized the importance of non-technical skills, referred to as soft-skills in the literature (Neubert et al. 2015). These non-technical skills are required in two main areas: (1) to understand customer demands and (2) to relate the technical aspects of jobs in terminologies for budgeting and decision-making purposes.

The findings of this study have direct implications for business schools. First, changes in job structures directly influence the type and level of skills demanded by employers. The mismatch between business graduates’ capabilities and employers’ expectations may limit initial employment and career progress (Dolton and Silles 2008; McGuinness and Sloane 2011). It is necessary for education institutions to consider job changes (structural due to technology when designing education programs). Still, U.S. higher education institutions have largely remained the same over the last decades, as curricula are designed by the academic world, with limited practitioner input. It is important that the skills taught and the means by which education is delivered be aligned with changes in job structures. Environments should be provided that encourage creativity and innovation in the classroom. Students graduate into work that requires problem solvers with the abilities to evaluate situations at a systems level and collaborate with others on how to continually improve their organization. The challenge for business schools and higher education institutions as a whole are to provide students with the exposure to develop these skills. No doubt, there needs to be increasing ties between education institutions and employers.

With the growth in the non-traditional student populations, those that are less inclined to return to school on a full-time basis, but are increasingly participating in job training, raise the issue of incorporating or developing means of certifying experiences gained at work. Job experience has always been recognized by employers; thus, education institutions should consider developing universal measures for credentialing work-based training. These changes should not be viewed as a threat to traditional higher education institutions, but as opportunities to reach a wider population of students and as a way to collaborate with employers to re-engineer the education system to meet the realities of the 21st-century workplace.

Business schools, in particular, are to prepare future managers and leaders, not just with skills to work within a company or industry but with transferable ones for the changing labor market. This study further affirms previous research on the skills business graduates require, such as critical thinking, negotiating, politicking, and communicating. Knowledge of typical business functions, such as accounting, finance, management information systems is still paramount, but there needs to be an integration of the non-technical skills within the curricula.

This study provides the background for a more extensive qualitative research, using a larger sample of college graduates, from a variety of institutions and non-business fields of study. It could be argued that the encroachment of technology on business professions, as described in this paper is less expected in comparison to more technology intensive jobs. Nevertheless, this area of study helps to inform stakeholders interested in the role and long-term existence of business schools.
